# Impaired Testicular Function without Altering Testosterone Concentration Using an Anti-Follicular-Stimulating Hormone Receptor (Anti-FSHr) Single-Chain Variable Fragment (scFv) in Long-Tailed Macaques (*Macaca fascicularis*)

**DOI:** 10.3390/ani13142282

**Published:** 2023-07-12

**Authors:** Pakpoom Navanukraw, Sroisuda Chotimanukul, Taratorn Kemthong, Kiattawee Choowongkomon, Kaywalee Chatdarong

**Affiliations:** 1 Department of Obstetrics, Gynecology and Reproduction, Faculty of Veterinary Science, Chulalongkorn University, Bangkok 10330, Thailand; pakpoomnavanukraw@gmail.com (P.N.); sroisuda.c@chula.ac.th (S.C.); 2 National Primate Research Center of Thailand, Chulalongkorn University, Saraburi 18110, Thailand; taratorn.k@chula.ac.th; 3 Department of Biochemistry, Faculty of Science, Kasetsart University, Bangkok 10900, Thailand; kiattawee.c@ku.ac.th

**Keywords:** contraceptive, monkey, nanobody, phage display, single-domain antibody

## Abstract

**Simple Summary:**

The overlapping presence of free-ranging long-tailed macaques (*Macaca fascicularis*) and humans in tropical and southeast Asian countries, for example, Thailand and Indonesia, has resulted in instances of physical harm, vandalism, and potential transmission of zoonotic diseases. The development of contraception in the free-ranging long-tailed macaques is aimed at maintaining testosterone production to keep the social hierarchy and dominance behavior. Using phage-display library technology, this study developed a novel form of contraceptive for the long-tailed macaques, consisting of small antibody fragments called single-chain variable fragments (scFvs) against the extracellular domain of the follicular-stimulating hormone receptor (anti-FSHr). The purity, size, and binding efficiency of the anti-FSHr scFv were characterized. Then, the study evaluated the effects of intratesticular administration of the anti-FSHr scFv on testicular function and serum testosterone levels in three adult macaques. The results indicated that the anti-FSHr scFv reduced sperm output without altering testosterone levels. This study provides promising insights into the development of new methods of contraception for long-tailed macaques.

**Abstract:**

FSHr antibodies have been shown to inhibit the differentiation of spermatogonia to primary spermatocytes, resulting in infertility without a pathological effect on reproductive organs. The aim of this study was to develop single-chain variable fragments (scFvs) against the follicular-stimulating hormone receptor (anti-FSHr) using phage-display technology and to evaluate the effects of intratesticular administration of the anti-FSHr scFv on testicular function and testosterone production. A phage clone against the extracellular domain of FSHr selected from a scFv phagemid library was analyzed for binding kinetics by surface plasmon resonance. Using ultrasound guidance, three adult macaques (*M. fascicularis*) were administered with 1 mL of 0.4 mg/mL anti-FSHr scFv (treatment) and 1 mL sterile phosphate buffer solution (control) into the left and right rete testis, respectively. Testicular appearance and volume, ejaculate quality, and serum testosterone levels were recorded on day 0 (before injection) and on days 7, 28, and 56 (after injection). Testicular tissue biopsies were performed on day 7 and day 56 to quantify the mRNA expressions of androgen binding protein (*ABP*), inhibin subunit beta B (*IHBB*), and vascular endothelial growth factor A (*VEGFA*). The results demonstrated that the anti-FSHr scFv molecule was calculated as 27 kDa with a dissociation constant (K_D_) of 1.03 µM. The volume of the anti-FSHr scFv-injected testicle was reduced on days 28 and 56 compared with day 0 (*p* < 0.05). Total sperm number was reduced from day 0 (36.4 × 10^6^ cells) to day 56 (1.6 × 10^6^ cells) (*p* < 0.05). The percentage of sperm motility decreased from day 0 (81.7 ± 1.0%) to day 7 (23.3 ± 1.9%), day 28 (41.7 ± 53.4%), and day 56 (8.3 ± 1.9%) (*p* < 0.05). Sperm viability on day 0 was 86.8 ± 0.5%, which reduced to 64.2 ± 1.5%, 67.1 ± 2.2%, and 9.3 ± 1.1% on days 7, 28, and 56, respectively (*p* < 0.05). The expression of *ABP* and *VEGFA* on days 7 (14.2- and 3.2-fold) and 56 (5.6- and 5.5-fold) was less in the scFv-treated testicle compared with the controls (*p* < 0.05). On day 56, the expression of *IHBB* was less (*p* < 0.05) in the treated testis (1.3-fold) compared with the controls. Serum testosterone levels were unchanged throughout the study period (*p* > 0.05). This study characterized the anti-FSHr scFv and demonstrated that treatment with anti-FSHr ameliorates testicular function without altering testosterone levels, offering a potential alternative contraceptive for the long-tailed macaques.

## 1. Introduction

The overlapping presence of free-ranging long-tailed macaques (*Macaca fascicularis*) and humans in tropical and southeast Asian countries, for example, Thailand and Indonesia, has resulted in physical harm, vandalism, and potential transmission of zoonotic diseases [1,2]. Because testosterone is obligatory for maintaining the normal physiology, muscle mass, body weight, aggression, and sexual behavior responsible for the male social hierarchy, research into the development of contraceptives for free-ranging macaques have been focused on maintaining testosterone production [3]. While testosterone is synthesized by the Leydig cells [4], in the testis, spermatogenesis, including cycles of spermatogonial self-renewal, germ-cell differentiation, meiosis, spermiogenesis, and spermiation, takes place in the seminiferous tubules [5,6]. Sertoli cells are somatic cells within the testis microenvironment that support male germ-cell development during spermatogenesis. The positive correlations between total Sertoli cell numbers and daily sperm production have been reported in several species [4,5]. This relationship exists because each Sertoli cell is able to sustain a limited number of germ cells [6]. Thus, it may be concluded that the appropriate development of the Sertoli cell population, with respect to their number and functionality, would determine the spermatogenic capacity throughout the lifespan [6,7].

As a contraceptive vaccine, FSHr antibodies have been shown to inhibit the differentiation of spermatogonia to primary spermatocytes, resulting in induced infertility without pathological effects on reproductive organs [8,9]. However, there are other glycoprotein hormone receptors (GPHr), e.g., luteinizing hormone receptor (LHr), human chorionic gonadotrophin receptor (hCGr), and thyroid-stimulating hormone receptor (TSHr), that are structurally similar to FSHr. To avoid interfering with other related GPHrs, an extracellular NH2-terminal domain (ECD) of the FSHr protein is selected for antibody development [10,11]. The ECD of FSHr has lower homology (39% to TSHr and 46% to LHr/CGr) compared with the intracellular transmembrane domain (68% homology to TSHr and 72% to LHr/CGr) [12]. In mice, antibodies against the ECD prevented fertility by inhibiting FSHr signal transduction, resulting in a decrease in cyclic adenosine monophosphate (cAMP) [13,14]. Consequently, the signal inhibition contributes to reductions in androgen binding protein (ABP) levels and inhibin synthesis [15]. However, producing such a large-sized full-length antibody (approximately 150 kDa) is laborious, time-consuming, and requires mammalian expression systems [16]. These limitations have led to the development of small antibody fragments (25 to 30 kDa) known as single-chain variable fragments (scFvs) to replace the full-length antibody.

The scFv consists of variable regions of heavy (*VH*) and light (*VL*) chains joined by a peptide linker [17]. It functions as the antigen-binding domain of an antibody, providing several advantages beyond conventional monoclonal antibodies. Firstly, scFvs are produced rapidly in microbial systems at low cost [18]. Their small size also enhances tissue distribution and penetration, contributing to improved pharmacokinetics [19]. Furthermore, scFvs have high specific-binding affinities that can be produced in bacterial cells and do not require testing in laboratory animals [20]. Using phage-display technology, a scFv is fused with bacteriophage-coated protein to bind with the target of interest [21]. Thereafter, specifically bound phages displaying scFvs are amplified by the infection of *Escherichia coli* [17]. ScFvs to stimulate and inhibit signaling pathways have been constructed in previous studies against glycoprotein hormone receptors, including luteinizing hormone receptors (LHrs) and the parathyroid hormone receptor (PTHr) [22,23].

This study aimed to (1) construct and characterize anti-FSHr scFvs developed from a human scFv phagemid library (Tomlinson I + J) using phage-display technology, and (2) to evaluate the effects on testicular function and serum testosterone levels after intra-testicular injection in long-tailed macaques (*M. fascicularis*).

## 2. Materials and Methods

### 2.1. Development of Anti-FSHr scFvs

#### 2.1.1. Selection of Anti-FSHr scFvs by Phage Library Biopanning

**Figure 1:**
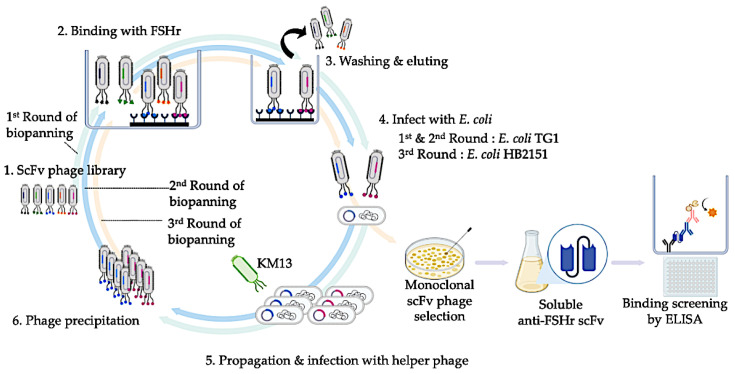
Schematic illustration of anti-FSHr scFv. In the first round, the Tomlinson I + J phage-display library was incubated with the ECD of the FSHr protein. Unbound phage were removed using washing buffer and eluted. The FSHr bound to phage were eluted and used to infect *E. coli* TG1 grown on LB agar plates. Each monoclone was propagated by infection with KM13 helper phage to display scFvs. The phage from the first round were subjected to the second round of biopanning. The phage from the second round were subjected to the third round of biopanning. The FSHr-bound phage from the third round were eluted and used to infect *E. coli* HB2151. Soluble anti-FSHr scFvs were retrieved from monoclonally infected *E. coli* HB2151 colonies. The scFv phage binding to FSHr was screened by the ELISA technique and selected purification and binding kinetic analysis. Created with Biorender.com.

A biopanning process was performed using the purified macaque ECD of the FSHr protein as a target according to the previous study [24]. A 96-well microtiter plate (Thermo Fisher Scientific, Waltham, MA, USA) was coated with 5 μg FSHr protein and incubated at 37 °C for 16 h. Excess FSHr target was removed by washing with 0.05% Tween-20 in phosphate buffer solution (PBS). The wells were blocked with 5% skim milk in PBS at 4 °C for 1 h and washed with 0.05% Tween-20 in PBS. Then, 100 μL of phage (1 × 10^10^) from a human scFv phagemid library (Tomlinson I + J) was added into the wells and incubated at 4 °C for 1 h for phage enrichment. Afterwards, unbound phage were removed by washing with 0.5% Tween-20 in PBS. The FSHr-bound phage were eluted by 100 μL of 0.2 M Glycine-HCl pH 2.2 and incubated for 15 min. Then, 15 μL of 1 M Tris-HCl pH 9.1 was added into the solution for neutralization. Mid-log phase *E. coli* TG1 were infected with the bound phage. The phage-infected *E. coli* TG1 was cultured on Luria-Bertani (LB) agar containing 100 μg/mL ampicillin. The KM13 helper phage was infected into bacterial cells harboring the phagemid and incubated at 37 °C for 1 h. The supernatant of *E. coli* TG1 infected with the KM13 helper phage was precipitated using PEG precipitation by adding 20% polyethylene glycol in 25 M NaCl and incubating on ice for 1 h. The phage precipitate was then centrifuged at 3200× *g* for 30 min. The precipitated phage pellets from the first round of biopanning were collected, resuspended in PBS, and filtered through a 0.45 μm Nylon syringe filter. The phage from the first round, instead of the phage library, were subjected to the next round. The second round of biopanning was performed as previously described and the precipitated phage pellets from the second round were subjected to the third round as illustrated in Figure 1.

To retrieve soluble anti-FSHr scFvs, twenty-four scFv samples from *E. coli* TG1 from the third round of biopanning were randomly selected. The phagemid from each of these colonies was rescued by KM13 helper phage and precipitated by PEG as described above. The individual phage were used to infect a non-suppressor *E. coli* HB2151 strain. Briefly, *E. coli* HB2151 in mid-log phase were infected with the individual phage clones. The infected *E. coli* HB2151 cells were cultured at 37 °C in 3 mL of LB medium containing 100 μg/mL of ampicillin to reach OD600 = 0.5. Then, clones containing the expressed plasmids were induced for soluble scFv production under IPTG induction. Isopropyl β-D-thiogalactopyranoside (IPTG) was added to reach a final concentration of 1 mM and incubated overnight at 25 °C. After incubation, bacterial cells were collected by centrifugation at 4 °C, 3200× *g* for 15 min. Supernatants containing anti-FSHr scFvs were collected for the ELISA.

#### 2.1.2. Binding Screening of FSHr Protein Bound-scFv Clones by Indirect Enzyme-Linked Immunosorbent Assay (ELISA)

Indirect ELISA was performed for binding screening as described in the previous protocols [24]. The macaque ECD of the FSHr protein (50 μL; 5 μg) was immobilized on a 96-well microtiter plate and incubated at 37 °C overnight. Bovine serum albumin (BSA) was used as a control. Excess protein was removed by 200 μL of washing buffer (1× PBS pH 7.4). Then, 200 μL of blocking buffer (3% BSA in 1× PBS pH 7.4) was added for 1 h. The supernatant containing soluble scFv from the *E. coli* HB2151 colonies was added to FSHr protein-immobilized wells and incubated for 1 h. The cell lysate of *E. coli* HB2151 in PBS was used as a negative control.

**Figure 2:**
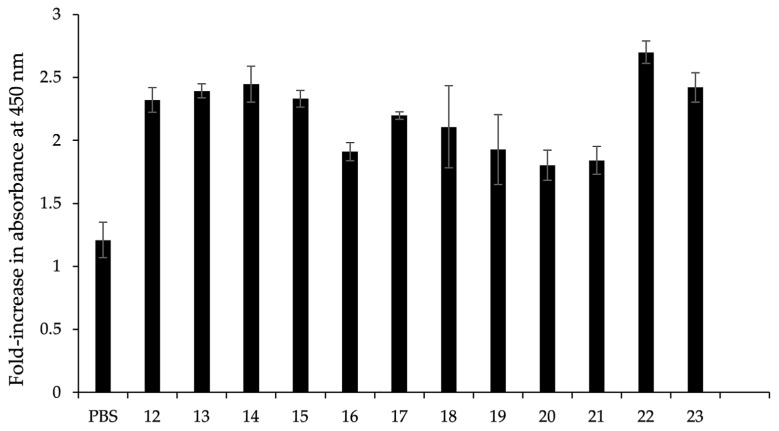
Fold-increase in optical density at wavelength 450 nm (A450) of indirect ELISA binding interaction to the FSHr protein between scFvs from bacterial colonies and bovine serum albumin (BSA). Error bars indicate ± SEM of triplicate experiments. Colony no. 22 was chosen for the further purification and binding kinetic determination.

For detection, the c-Myc monoclonal antibody (1:3000, Thermo Fisher Scientific, Waltham, MA, USA) and horseradish peroxidase (HRP)-conjugated goat-anti mouse antibodies (1:5000, Thermo Fisher Scientific, Waltham, MA, USA) were used as the primary and secondary antibody, respectively. To indicate the binding reaction of scFv to the FSHr protein from individual phage, 100 μL TMB (Life technologies, Carlsbad, CA, USA) with 1% of H_2_O_2_ as the substrate for HRP was added, and the mixture was incubated in the dark at room temperature. Lastly, 100 μL of 1 M of hydrogen chloride (HCl) was added to cease the peroxidation reaction. Reactions were determined by measuring the absorbance at wavelength 450 nm (A450) using an ELISA plate reader. Colony no. 22 exhibited the highest binding interaction among all the colonies, which was 2.7-fold greater compared with binding to BSA. Colony no. 22 was chosen for the further purification and binding kinetic determination (Figure 2).

#### 2.1.3. Anti-FSHr scFv Expression and Purification

The supernatant of colony no. 22 was purified by using Ni-NTA Affinity Resin—Amintra (Expedeon, Cambridge, UK)) as described previously [19]. The elution throughout the purification process was analyzed for purity and size by 12% sodium dodecyl sulfate polyacrylamide gel electrophoresis (SDS-PAGE), with bands observed by Coomassie blue staining [24].

### 2.2. Kinetics and Affinity Determination by Surface Plasmon Resonance (SPR)

Binding kinetics of the anti-FSHr scFv was determined by SPR (OpenSPR, Nicoya Lifesciences, Kitchener, ON, Canada). The ligand was activated for COOH groups by combining 100 μL of 0.030 mg/mL FSHr protein in 10 mM PBS pH 7.4 (running buffer), 50 μL of 0.2 M 1-Ethyl-3-(3-dimethylaminopropyl) carbodiimide (EDC), and 50 μL of 0.05 M N-hydroxysuccinimide (NHS) and incubating at 4 °C for 1 h [24]. All measurements were performed in running buffer. The NH_2_-sensor chip (Nicoya Lifesciences) was loaded into the OpenSPR instrument and baseline measurements were performed. Then, 200 μL of blocking solution was injected to inactivate non-immobilized scFv. Two-fold dilution concentrations of the anti-FSHr scFv (10, 5, 2.5, 1.25, 0.625 μg/mL) in 200 μL were injected at a flow rate of 20 μL/min for both association and dissociation measurements. Kinetic data were analyzed by Trace Drawer version 1.6.1 using the 1:1 binding model, and the respective rate constant values were calculated.

### 2.3. Animals

Three adult long-tailed macaques, aged 6, 7.5, and 8.5 years and with a body weight of 6.8, 8.17, and 8.3 kg, respectively, were raised at the National Primate Center, Chulalongkorn University (NPRCT-CU). This facility has been approved by AAALAC International Accreditation. The long-tailed macaques were kept in individual stainless-steel squeeze-back cages (60 × 70 × 85 cm) in strictly hygienic conditions at 25 ± 2 °C and 60 ± 10% relative humidity. They were housed in a controlled environment with a photoperiod of 12 h of light and 12 h of darkness. This lighting schedule aimed to replicate the natural day–night cycle and was maintained consistently throughout the duration of the study. All macaques were fed with standard monkey chow (Perfect Companion Group Co., Ltd., Bangkok, Thailand) and fresh tropical fruits. Fresh hyperchlorinated water (1 ppm) was available ad libitum through an automatic lixit faucet. The macaques did not receive any antibiotic treatment and were not used for any experiment prior to this study. The experimental protocol was approved by the Chulalongkorn University Animal Care and Use Protocol (CU-ACUP) at the NPRCT-CU (Protocol review number: 2175011; Approval date: 17 November 2021). During the study, the macaques were clinically observed daily by a veterinarian and a veterinary technician. The observations included overall general appearance, posture, behavior, scrotal changes, scrotal pain, appetite, urination, defecation, and abnormal breathing patterns.

### 2.4. Intratesticular Administration

Feed and water were withheld for 12 h before the administration of 2 to 5 mg/kg tiletamine/zolazepam combined with 12.5 to 50 mg/kg dexmedetomidine intramuscularly through the squeezing cage. Once immobilized, the macaques were physically examined by attending veterinarians, who also performed a reproductive examination that included assessing testicular size, circumference and consistency and ultrasonography. Blood samples were collected from the femoral vein, followed by semen collection using rectal electroejaculation. All macaques were given intratesticular injections of anti-FSHr scFv (treatment) in one testis and sterile PBS (control) in the other testis.

For the intratesticular injections, the macaques were placed in the dorsal recumbency position. Aseptic technique of the scrotal sac was performed for surgical preparation with 70% ethanol and 10% povidone. The testes were securely held. Injections were given using 26-G ½″ needle attached to a 1 mL plastic disposable syringe introduced at the caudal area of the testis, lateral to the caudal epididymis. The needle inserted parallel to the testis into the rete testis under ultrasonographic guidance. A single injection of 1 mL anti-FSHr scFv (0.4 mg/mL) or PBS was slowly given into each testis (1 mL/min) [25]. The needle was removed slowly and steadily to avoid solution leakage. After finishing, animals were injected with atipamezole via the intramuscular route and allowed to safely recover in their cages.

General appearance, behavior, and scrotal changes (e.g., pain, swelling, dermatitis) were observed once daily after injection until the end of the study. The duration of this study was determined based on the length of the complete spermatogenesis cycle and epididymal sperm maturation, which is approximately 56 days [26,27]. The length, width, and depth of each testis was measured by a vernier caliper (Mitutoyo, Kawasaki, Japan) along with testicular circumference by measuring tape before treatment (day 0) and on days 7, 28, and 56 after injection. The testicular volume was calculated by testicular length × width × depth × 0.71 as described by [28]. Testicular ultrasonography was performed to identify any post-injection complications.

### 2.5. Semen Collection and Evaluation

Semen samples were collected by electroejaculation under anesthesia before treatment (day 0) and on days 7, 28, and 56 after injection as adapted from Zainuddin et al. [29]. Briefly, a lubricated rectal probe (1.4 cm diameter with three longitudinal electrodes) was inserted approximately 4 to 7 cm into the rectum with the electrode directed ventrally and connected to an electro-ejaculator (Yenwa, Bangkok, Thailand). The electrical stimulus was applied for approximately 3 s at a 2 s interval. A series of electrical stimuli was initiated at 3.5 volts (V) (50 stimulations), gradually increased to 4 V (50 stimulations), and 4.5 V (50 stimulations) respectively. The highest voltage of 5 V was introduced in the case where no ejaculation was observed. Symmetrical muscle contractions and thigh extension were observed to ensure optimal probe positioning. Ejaculates containing liquid and coagulated fractions were collected into sterile specimen tubes (5 mL) and incubated at 37 °C for 30 min for liquefaction.

Semen appearance and consistency were assessed subjectively, including an examination of their color (colorless, yellowish, or whitish), opacity (opaque or transparent), and appearance (amorphous or filamentous seminal coagulum). The liquified portion was aspirated from the coagulum and transferred into a sterile 1.5 mL Eppendorf tube with a micropipette to determine the volume. The percentage of motile sperm and progressive motility were then evaluated using a phase-contrast microscope at 200× magnification (Olympus, Shinjuku, Japan) by 3 independent evaluators. Progressive motility was graded on a scale of 0 to 5 (0 = no forward progression, 5 = rapid forward progression) as previously described [30]. Sperm concentration was evaluated by diluting the sperm samples with formal saline using a hemocytometer (Boeco, Hamburg, Germany). Total sperm number per ejaculate was calculated by multiplying the sperm concentration with the total liquified volume. To assess sperm viability, samples were stained using eosin–nigrosin and 200 spermatozoa per sample were examined under a light microscope at 1000× magnification [31]. Five hundred sperm were evaluated for head morphology, while the sperm midpiece and tail morphology were evaluated by counting 200 sperm in formal-saline-diluted samples under a light microscope at magnification 1000× [32].

### 2.6. Testicular Biopsy

Testicular tissue samples were collected from both testes on days 7 and 56 from all subjects for quantitative polymerase chain reaction (RT-qPCR) analysis. A scrotal stab incision was performed in the caudal region, then a 16 G semi-automatic biopsy needle (MEDAX, San Possidonio, Italy) was inserted towards the center of the testicle. Testicular tissues were collected with a 10 mm notch. The tissue samples were kept in Trizol reagent and cryopreserved at −20 °C until RNA extraction.

### 2.7. Reverse-Transcription Polymerase Chain Reaction (RT-PCR) for Sertoli Cell Markers

#### 2.7.1. RNA Isolation and Reverse Transcription

Total cell RNA was isolated from frozen testicular tissues with the Direct-zol™ RNA MiniPrep Kits (Zymo Research, Irvine, CA, USA) according to the manufacturer’s instructions. Tissues were homogenized by a homogenizer at 5000 rpm for three cycles of 45 s with 15 s intervals (Minilys, Bertin Technologies, Montigny-le-Bretonneux, France) in homogenizing tubes (Tissue grinding CKMix 2 mL, Bertin Technologies). The RNA was finally eluted in 30 mL of pre-warmed (50 °C) RNase-free water. Its concentration and purity were determined by an ND-1000 Nanodrop spectrophotometer (Wilmington, DE, USA). Subsequently, the RNA was reversed transcribed into complementary DNA (cDNA) using the ImProm-II™ Reverse Transcription System (Promega, Madison, WI, USA).

#### 2.7.2. Primer Sequences and Optimization

Oligonucleotide primer sequences were newly designed (Table 1). For the optimization of the qPCR, primers were tested by conventional PCR amplification using the GoTaq^®^ Green Master Mix (Promega, Madison, WI, USA). The presence of a single band of DNA by electrophoresis on a 0.1% (*w*/*v*) agarose gel confirmed the specificity of the PCR products and the lack of primer dimers.

#### 2.7.3. Real-Time qPCR

Expression levels of the Sertoli cell function-related genes androgen binding protein (*ABP*), inhibin subunit beta B (*IHBB*) and vascular endothelial growth factor A (*VEGFA*) and the reference gene glyceraldehyde-3-phosphate dehydrogenase (*GAPDH*) were determined by the absolute real-time qPCR method (CFX96 Real-Time PCR Detection System, Bio-Rad Laboratories, Inc., Hercules, CA, USA). For the qPCR reaction, 2 μL diluted cDNA samples, each equivalent to 10 ng of total RNA, were analyzed in a 25 μL reaction volume using a master mix (KAPA SYBR FAST qPCR, KAPA Biosystems, Wilmington, MA, USA) and 10 μM each of the appropriate reverse and forward primers. The qPCR reactions were run in triplicate. Data were evaluated in enclosed PCR strip tubes (Bio-Rad Laboratories, Inc., Hercules, CA, USA). A non-template control using nuclease-free water was included for every primer pair to detect DNA contamination.

The qPCR conditions were: initial denaturation at 95 °C for 3 min, followed by 40 cycles of denaturation at 95 °C for 3 s, annealing at 60 °C for 20 s, and extension at 72 °C for 20 s. Melting curve analysis was performed from 65 °C to 97 °C in 0.5 °C steps, each lasting 10 s. The data were generated using CFX ManagerTM Software v. 3.1 (Bio-Rad Laboratories, Hercules, CA, USA). The absolute copy number of the PCR product was determined by comparing the CT values of the unknown sample to a standard curve using the Bio-Rad CFX Manager software version 3.1.

### 2.8. Testosterone Assay

Blood samples were collected from femoral venipuncture before treatment (day 0), and on days 7, 28, and 56 after treatment. The samples were centrifuged (700× *g* for 15 min), and sera were stored at −20 °C. Serum testosterone concentrations were measured in one batch at the Endocrine Laboratory, Conservation Research and Animals Health, Khao Kheow Open Zoo (Chonburi, Thailand). Testosterone polyclonal testosterone antibody (R156/7), testosterone-horseradish peroxidase (C.J. Munro, University of California, Davis, CA, USA) and testosterone standards (Steraloids, Inc., Newport, RI, USA) were used for the assay. The assay sensitivity was 0.05 ng/mL, and the intra-assay CV was 7.58%.

### 2.9. Statistical Analysis

All data were presented as mean ± SEM and were analyzed using the SPSS Statistics for Windows, Version 22.0 (Armonk, NY, USA: IBM Corp). A paired t-test was performed to compare differences in the means of testicular measurements, sperm quality parameters, testosterone levels, and gene expression levels. The means were considered significant when *p* < 0.05.

## 3. Results

### 3.1. Characterization of Anti-FSHr scFv

**Figure 3:**
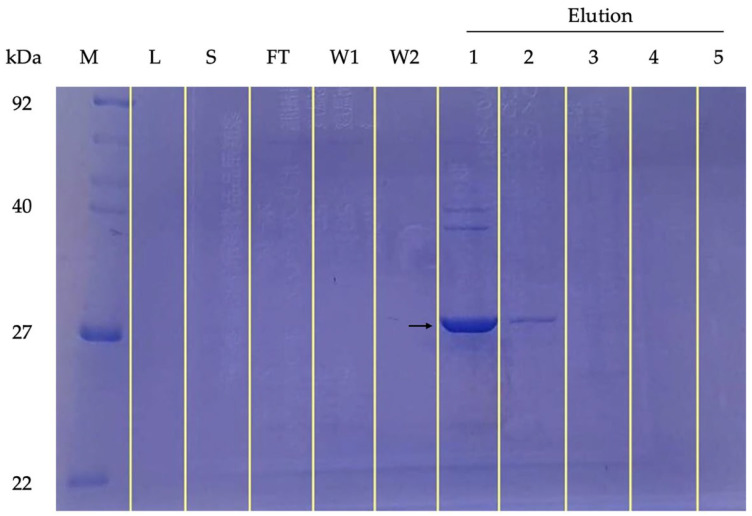
SDS-PAGE analysis of purified anti-FSHr scFv. The arrow indicates anti-FSHr scFv from elution 1 (27 kDa). (Marker: protein marker; Medium: LB medium; S: supernatant; FT: flow-through; W1: washing buffer 1; W2: washing buffer; Elution 1−5: scFv fraction eluted 1–5 rounds by imidazole).

The constructed anti-FSHr scFv was expressed and purified from bacterial colony no. 22 in this study. The purity was demonstrated by the presence of a single band with a molecular weight of 27 kDa corresponding to the scFv protein in the first and second rounds of imidazole elution (Figure 3). No scFv protein bands were observed in the LB medium, supernatant, flow-through, and washing buffers.

**Figure 4:**
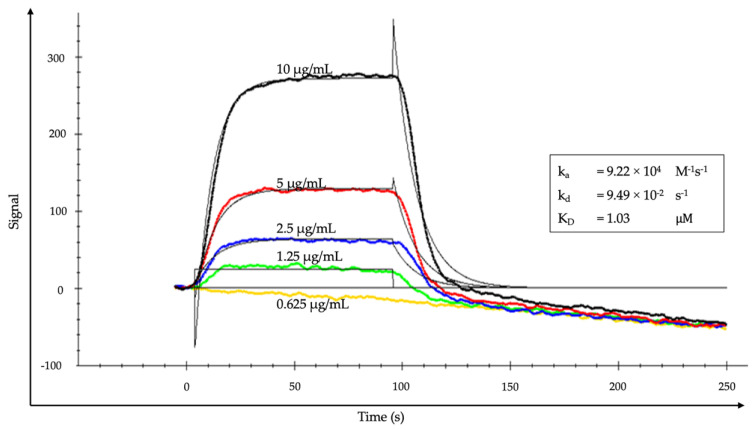
Surface plasmon resonance sensorgrams of different concentrations of anti-FSHr scFv binding to FSHr. The evaluation of anti-FSHr scFv gave k_a_ = 9.22 × 10^4^ M^−1^s^−1^, k_d_ = 9.49 × 10^−2^ s^−1^, and K_D_ = 1.03 µM. (k_a_: association rate constant; k_d_: dissociation rate constant; K_D_: equilibrium dissociation constant).

The SPR binding profiles are presented in Figure 4. The binding affinity of the anti-FSHr scFv indicated an equilibrium dissociation constant (K_D_) of 1.03 µM with an on-rate constant (k_a_) of 9.22 × 10^4^ M^−1^s^−1^ and an off-rate constant (k_d_) of 9.49 × 10^−2^ s^−1^.

### 3.2. Testicular Appearance

The general health as well as vital signs of the macaques were within normal limits. Testicular swelling was not evident after the injection on day 0. At the end of the study, both testicles were comparably normal and without inflammation. No complications were observed by ultrasonography after intratesticular injection nor testicular biopsy.

**Figure 5:**
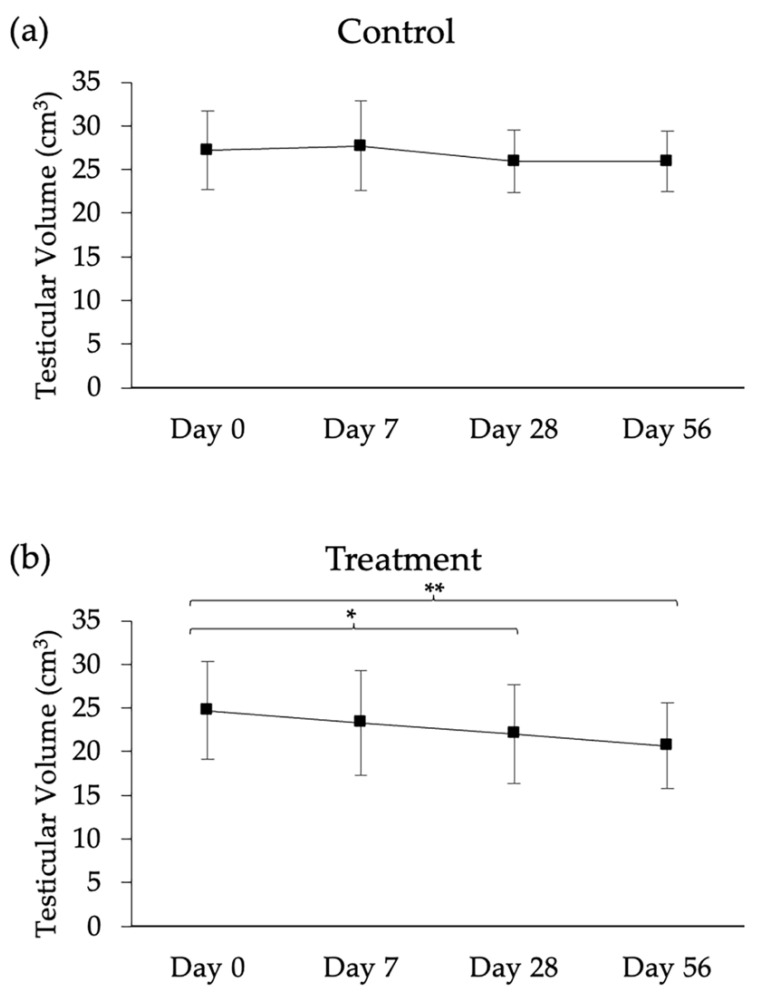
Testicular volume (mean ± SEM) of long-tailed macaques (*n* = 3) following intratesticular injection of 1 mL PBS into one testis ((**a**); control) and 1 mL anti-FSHr scFv (0.4 mg/mL) into the contralateral testis ((**b**); treatment). Compared with day 0, testicular volume was reduced on day 28 and day 56. The asterisk indicates a statistical difference (*, *p* < 0.05; **, *p* < 0.01).

The testicular volumes (control and treatment) throughout the study are presented in Figure 5. The mean testicular volume of the control testis was 22.8 ± 4.5 cm^3^, 28.2 ± 5.2 cm^3^, 26.0 ± 3.6 cm^3^, and 25.9 ± 3.6 cm^3^ on days 0, 7, 28, and 56 respectively. Changes in the volume of control testes were not significant between day 0 compared with days 7, 28, and 56 (*p* > 0.05). However, the mean testicular volume of the treated testis significantly reduced on day 28 (n1 = 29.7 cm^3^, n2 = 21.4 cm^3^, n3 = 11.0 cm^3^; *p* < 0.05) and day 56 (n1 = 27.1 cm^3^, n2 =24.3 cm^3^, n3 = 10.9 cm^3^; *p* < 0.01), compared with day 0 (n1 = 31.5 cm^3^, n2 = 29.0 cm^3^, n3= 13.2 cm^3^).

### 3.3. Semen Quality

The ejaculates contained liquid and coagulated seminal fractions. The liquid fractions were transparent and colorless. The coagulated fractions were opaque and white, and their appearance was either filamentous or amorphous.

**Figure 6:**
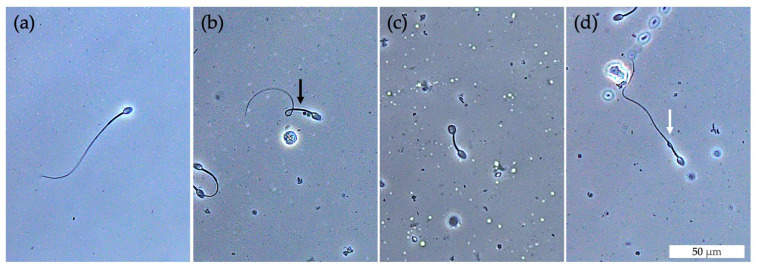
Light micrographs of normal and abnormal sperm tail morphologies of long-tailed macaques after intratesticular injection of anti-FSHr scFv on day 56. (**a**) Normal sperm. (**b**) Bent tail (black arrow). (**c**) Coiled tail. (**d**) Distal droplet (white arrow). Scale bar in (**d**) is applicable to all the micrographs.

Quantitative data for total sperm number as well as the percentages of sperm parameters are presented in Table 2. The total sperm numbers on days 0, 7, 28, and 56 were 36.4 ± 4.5, 15.1 ± 1.1, 9.5 ± 0.7, and 1.6 ± 0.2 × 10^6^ cells, respectively. Interestingly, the total sperm number on day 56 was significantly decreased compared with day 0 (*p* < 0.05). The percentage of sperm motility compared with day 0 was reduced on day 7 (*p* < 0.001), day 28 (*p* < 0.05), and day 56 (*p* < 0.001) (Table 2). The mean percentages of sperm viability on days 0, 7, 28, and 56 were 86.8 ± 0.5%, 64.2 ± 1.5%, 67.1 ± 2.2%, and 9.3 ± 1.1%, respectively. The percentage of sperm viability post-treatment on days 7 and 28 was significantly decreased compared with day 0 (*p* < 0.05) and demonstrated a greater decrease on day 56 (*p* < 0.001, Table 2). The percentage of sperm with normal tail morphology on days 7 and 56 was significantly lower than on day 0 (*p* < 0.05; Table 2). Most of the abnormal sperm tail morphology was bent tail followed by coiled tail and distal droplet. The pictures of normal and abnormal sperm tail morphologies are presented in Figure 6. However, there was no significant difference in sperm tail morphology between day 0 and day 28 (*p* > 0.05, Table 2). The percentage of morphologically normal sperm heads did not differ throughout this study (*p* > 0.05; Table 2).

### 3.4. Sertoli Cell Function and Related Gene Expression

**Figure 7:**
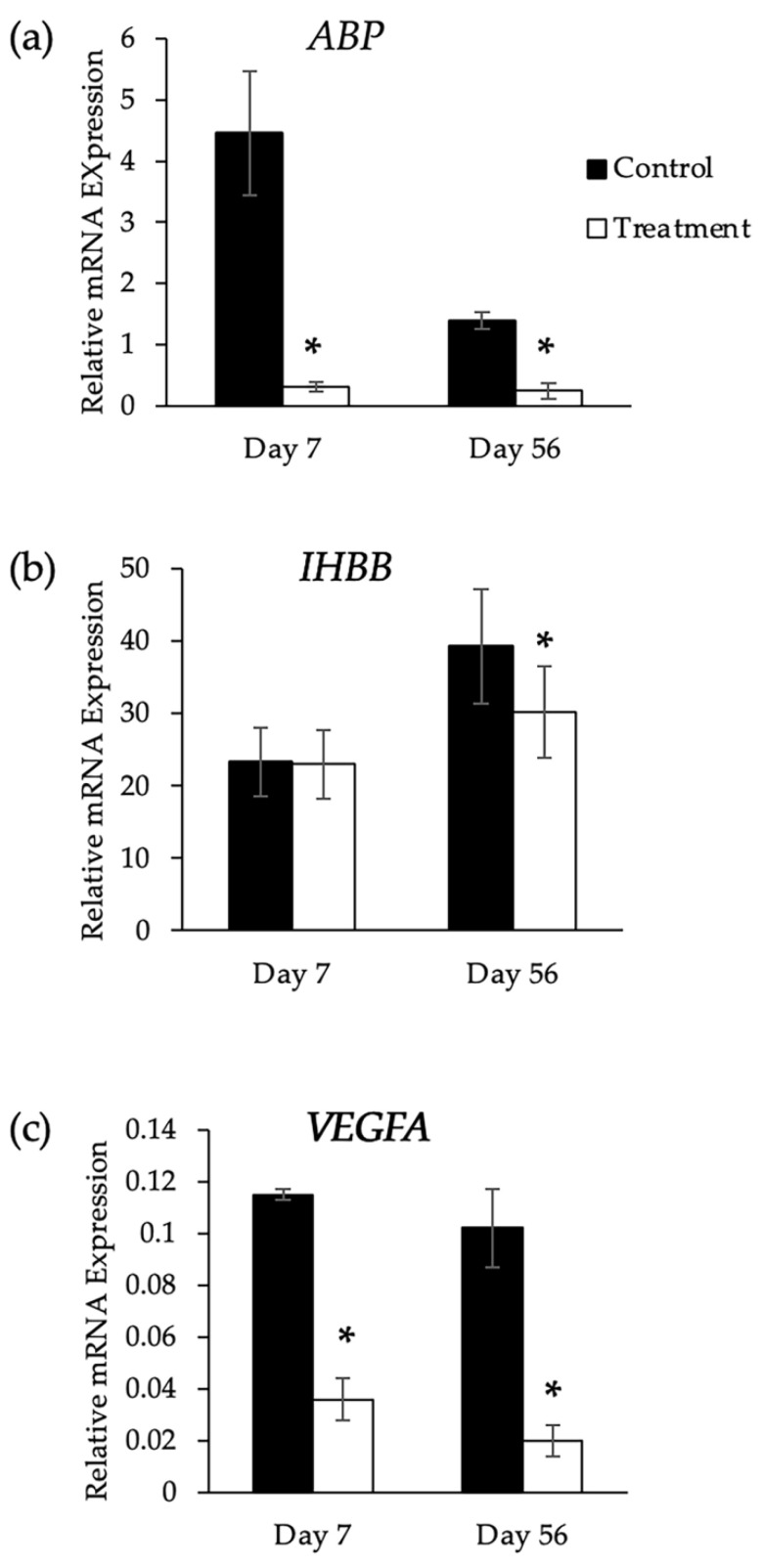
Comparison of gene expression in long-tailed macaques (*n* = 3) on days 7 and 56 after intratesticular injection of PBS (Control) in one testis and anti-FSHr scFv in the contralateral testis (Treatment). (**a**) *ABP*: androgen binding protein; (**b**) *IHBB*: inhibin subunit beta; (**c**) *VEGFA*: vascular endothelial growth factor A. Relative mRNA expression (arbitrary unit) was determined by qPCR and normalized against CYPA using the 2^ΔΔCT^ method. Asterisk (*) indicates statistical significance (*p* < 0.05).

The *ABP* relative expression on days 7 and 56 was lower in the treatment than the control tissues, being 14.2- and 5.6-fold less respectively (*p* < 0.05; Figure 7a). No difference in *IHBB* expression was seen on day 7 between the control and treatment groups (*p* > 0.05; Figure 7b). However, on day 56, *IHBB* was significantly less by 1.3-fold (*p* < 0.05, Figure 7b). The *VEGFA* mRNA expression on day 7 was 3.2-fold less (*p* < 0.05) in the treatment compared with the control tissues. Similarly, on day 56, the *VEGFA* mRNA expression was 5.0-fold less (*p* < 0.05; Figure 7c) in the treatment compared with the control tissues.

### 3.5. Serum Testosterone Levels

**Figure 8:**
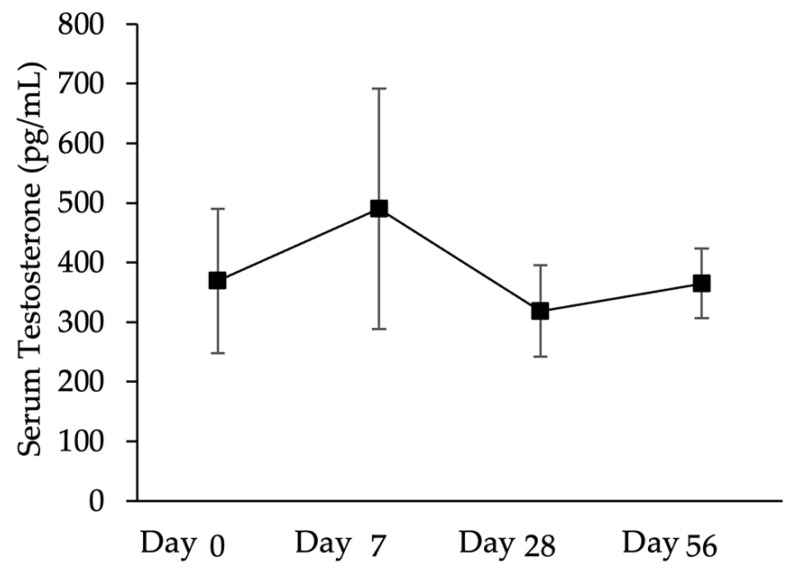
Serum concentration of testosterone (mean ± SEM) of long-tailed macaques (*n* = 3) on days 0, 7, 28, and 56 after intratesticular injection of 1 mL of 0.4 mg/mL scFv in one testis.

The serum testosterone concentrations did not differ (*p* > 0.05) over 56 days following the administration of anti-FSHr scFv (Figure 8). The mean concentration of testosterone was 369.3 ± 121.1 pg/mL on day 0, 490.4 ± 201.8 pg/mL on day 7, 318.4 ± 76.7 pg/mL on day 28, and 364.9 ± 58.1 pg/mL on day 56.

## 4. Discussion

### 4.1. Characterization of scFV Anti-FSHr

In this study, an scFv specific to the ECD of the FSHr was successfully constructed by phage-display screening from the phagemid library. ScFv fragments have been commonly produced against tumors for diagnostic and therapeutic purposes [33,34]. This method uses phage displaying a library of numerous scFv variants. These variants are screened against a target to identify ones with high affinity and specificity for the target of interest [31]. The inhibitory activity of the anti-FSHr scFv in this study was in accordance with the results in the previous study using FSHr recombinant antibodies [35]. The recombinant antibodies against FSHr from a synthetic phage library demonstrated the reduction of FSH-induced cAMP accumulation, indicating their inhibitory activity against the FSH signaling pathway, leading to FSHr inhibition [35].

In this study, the anti-FSHr scFv selected from the phage library promoted a significant reduction in sperm parameters, including sperm number, motility, and viability as well as Sertoli cell functions. The development of single-chain variable fragments (scFvs) represents a significant advancement in antibody engineering due to their small size and lack of a hinge region, which makes them superior to full-length antibodies in terms of production and efficacy [36]. With a size of 27 kDa, the scFvs produced in this study could be manufactured in bacterial cytoplasm and provided high and rapid yields for amplification, according to a previous report [18].

This study successfully purified an anti-FSHr scFv from *E. coli*, as demonstrated by the SDS-PAGE analysis. Without the protein hinge region, the scFv was likely to have increased solubility, reduced aggregation, and improved stability, thus making its purification easier [36]. Additionally, without a crystallizable (Fc) region, the scFvs could eliminate the risk of immune cell activation and local and humoral immunity, allowing the molecule to bind its target without activating the host immune response [34]. In relation to this study, no testicular swelling or inflammation was observed following the injection of anti-FSHr scFv into the rete testes of macaques, demonstrating the safety and efficacy of this approach.

In this study, the K_D_ value was in a micromolar range (1.03 µM), indicating moderate binding affinity between the anti-FSHr scFv and the ECD of the FSHr. The dissociation constant (K_D_) is an important parameter that characterizes the binding affinity of a protein for its ligand [37]. This range of K_D_ values was consistent with the previous report on scFv–LHr interactions [23]. However, developing a strong binding efficacy in the nanomolar range might be possible through the selection of different phage colonies. The selection process involves the screening of a large library of phage-displayed protein variants to identify the strongest binding affinity and optimizing conditions to enhance the binding properties [38]. Taken together, this study demonstrated the characteristics of an scFv against the ECD of the FSHr developed by phage-display technology. These results highlight the potential of an anti-FSHr scFv as a promising candidate for the development of novel contraceptives.

### 4.2. Anti-FSHr scFv Effects on Testicular Tissues

During spermatogenesis, germ-cell apoptosis increases when Sertoli cells are injured or experience a toxic environment [39]. The anti-FSHr injection in this study revealed the significant decrease in testicular volume on days 28 and 56 and the significant reduction in mRNA expression of ABP, IHBB, and *VEGF* in the treated testis compared with the control side, implying the possible loss of Sertoli cell functions. The reduction in the expression of these genes likely compromised vascular growth and the microenvironment required for the mitogenic activity of endothelial, vascular smooth muscle cells, and pericytes in testicular functions [40]. Thus, the decrease in testicular size was likely due to decreased activity of Sertoli cells and neighboring cells’ proliferation. The injection of anti-FSHr scFv is unlikely to have induced damage to the testicular tissues. This is in contrast to zinc-based solutions, for which adverse effects, including tissue necrosis, necrotizing orchitis, scrotal ulcerative dermatitis, and testicular abscess, associated with intra-testicular injection have been reported [41,42]. Moreover, zinc-gluconate injection induced testicular degeneration and disruption of spermatogenesis, leading to azoospermia and a reduction in testosterone synthesis [43]. The specific binding of the anti-FSHr scFv to FSHr antagonized its effects without causing post-injection complications. This highlights the potential of anti-FSHr scFv as a safe and effective male contraceptive.

In this study, the anti-FSHr was injected directly into the rete testis using ultrasonographic guidance to maximize its effect and distribution among the seminiferous tubules, following a previous suggestion [25]. With blood–testis barrier (BTB), the anti-FSHr scFv was assumed to be physiologically confined to the injected testis [44,45]. The BTB prevents most substances from passing between the blood circulation and the adluminal compartment of the testis by the tight junctions [45]. They control the movement of protein molecules between the compartments, creating a unique microenvironment for germ-cell development and maturation in the testis [46]. Thus, the anti-FSHr scFv solution was likely not transported across the BTB to affect the contralateral side.

### 4.3. Anti-FSHr scFv Effects on Semen Production and Sperm Morphology

This study revealed a significant decrease in testicular volume, which correlated with impaired sperm parameters, including total sperm number, motility, viability, and tail morphology. These findings were consistent with a previous report demonstrating the relationship between testicular volume and sperm parameters, including seminal fluid volume, concentration, motility, and morphology [47]. The reduced testicular volume of the anti-FSHr scFV-injected side, along with the reduced sperm number, reflected compromised testicular functions [48,49]. Testicular volume reflects the activity of seminiferous tubules that contain germ and Sertoli cells responsible for sperm production [48].

In this study, the total sperm number tended to decrease on days 7 and 28 and significantly reduced on day 56, suggesting that anti-FSHr scFv injection negatively affected germ cell development at all stages, as well as the ejaculated spermatozoa. Furthermore, the number of sperm with abnormal tails was increased, probably because of the impaired Sertoli cells. Generally, germ-cell differentiation is regulated using the energy supplied by Sertoli cells through lactate synthesis during spermatogenesis [50]. An energy deficit contributes to an increase in germ-cell apoptosis [39]. Thus, the metabolic activity of Sertoli cells plays a crucial role in regulating the activity of spermatogenesis.

In addition, our findings of the reductions in sperm motility, progressive motility, and viability inferred the impairment of Sertoli cells. Sertoli cells are involved in multiple cellular changes supporting spermatogenesis by providing nutritional support that leads to the formation of motile cells [51,52]. Suppression of Sertoli cell functions can negatively affect the overall motility and viability of the sperm due to inadequate nutrition and abnormal differentiation [51].

### 4.4. Anti-FSHr scfv Effects on Related Gene Expression

In this study, the lower *ABP*, *IHBB*, and *VEGFA* expression in the scFv-injected testis than the controls confirmed the antagonistic effect of the anti-FSHr scFv on the FSHr. *ABP* is a major carrier protein that delivers testosterone to the epididymis. It specifically binds to testosterone or dihydrotestosterone to reduce testosterone lipophilicity and increase their concentration in the seminiferous tubules [53]. The levels of ABP are necessary for maintaining the microenvironment, spermatogenesis in the seminiferous tubules, and sperm maturation in the epididymis. When ABP migrates to the caput epididymis, it facilitates the access of testosterone to support immature sperm and to facilitate sperm maturation [54]. Moreover, the ABP–androgen complex is also involved in other epididymal functions, including developing motility and fertilization capabilities [55]. The reduction in *ABP* expression, along with lower sperm motility post-treatment, clearly demonstrates the role of the FSHr signaling cascade in regulating ABP production, which affects epididymal functions and sperm motility [56].

IHBB, which is synthesized by Sertoli cells, serves as a circulating biomarker for evaluating the status of Sertoli cell function. Acting as a regulator in the hypothalamic–pituitary–testis axis, IHBB provides negative feedback for FSH secretion [57]. In the previous study, IHBB concentrations were found to be associated with spermatogenesis markers examined by semen analysis and testicular histology [58]. The suppression of *ABP* and *IHBB* mRNA expression along with the reduction in total sperm number in our study suggest compromised testicular functions.

This study has demonstrated that the anti-FSHr scFv treatment reduced *VEGFA* expression in the testicular tissues. VEGFA is a signaling polypeptide that has a crucial function in microvascular development by promoting endothelial cell mitogenesis and increasing microvascular permeability [59]. VEGFA improves sperm motility and viability by providing an adequate microenvironment for spermatogonial stem cells and spermatogenesis throughout its biological effect on the testicular microvasculature, with a greater density of capillaries providing increased delivery of gonadotropins to the developing sperm [60]. Therefore, a reduction in VEGFA in testicular cells may affect genes necessary for the maintenance of undifferentiated spermatogonia, eventually reducing sperm viability and the resulting male reproductive lifespan and fertility [61,62].

### 4.5. Anti-FSHr scfv Effects on Testosterone Levels

This study demonstrated the anti-FSHr scFv’s detrimental effect on spermatogenesis in the testes, with reference to the stabilization of testosterone levels in long-tailed macaques. Testosterone levels did not differ between time points throughout this study. Novel contraceptive approaches that do not disrupt steroidogenesis have become an optimal goal, not only in primates but also in companion animals, to avoid long-term health complications [63]. Gonadectomy in companion animals ceases the steroidogenesis of the hypothalamic–pituitary–gonadal axis, resulting in supraphysiological circulating LH concentrations due to reduced negative feedback [64]. The LH receptors are also present in non-reproductive tissues [57], and high LH concentrations can lead to health complications such as urinary incontinence, diabetes mellitus, and hypothyroidism, as well as neoplastic complications [63]. Therefore, developing human male contraceptives that do not disrupt testosterone levels is crucial for maintaining overall health and well-being. Testosterone levels are obligatory for maintaining libido and secondary sexual characteristics [65]. The anti-FSHr scFvs specifically target the ECD of the FSHr on Sertoli cells without interfering with Leydig cell steroidogenesis or testosterone levels, making them a promising candidate for further male contraceptive research.

Due to the small numbers of macaques in this study, we realize that there is the possibility of type II errors occurring, that is, the acceptance of a false null hypothesis (declaring no difference between treatments when a difference does exist), and this must be considered whenever small numbers of animals are assigned to the treatment group. Although our data highlight sperm number reduction with an injection of an anti-FSHr scFv, the specific role of FSHr scFvs and their applications warrant further safety studies.

## 5. Conclusions

The single injection of an anti-FSHr scFv into the rete testes of macaques appeared to cause disruption of the Sertoli cells, resulting in altered testicular functions. However, the serum testosterone was not affected. The anti-FSHr scFv is likely to be an efficacious and safe contraceptive in male long-tailed macaques. Our platform can likely be applied in free-ranging macaques or as a non-surgical sterilization in companion animals for population control.

## Figures and Tables

**Table 1:** Oligonucleotide primer sequences, GenBank accession, expected amplicon size, annealing temperature.

Gene #.	Primer Sequence (5′-3′ Orientation)	GenBank Accession	Product Length (bp)	Annealing Temp (°C)
*ABP*	FP: GCACATGACATACACAATCTT RP: GGGTTGGTATCCCCATAAAAA	XM_015437363.1	110	60
*IHBB*	FP: CTCCCCTTAGGTTCTGTTTC RP: CGTGGCACTCAATCTTTTAT	XM_005573058.2	116	60
*VEGFA*	FP: GTACATCTTCAAGCCATCCT RP: GAACGCTCCAGGACTTAT	NM_001110502.1	136	60
*GAPDH*	FP: CTCTGGGCGCATCCC RP: CTTGAGGCTGTTGTCATACT	XM_015430282.1	459	60

**Table 2:** Sperm parameters (mean ± SEM) in long-tailed macaques (*n* = 3) before and after intratesticular injection of anti-FSHr scFv (0.4 mg/mL) in PBS (1 mL) on days 0, 7, 28, and 56. Letters indicate significant differences (*p* < 0.05).

	Pre-Treatment	Post-Treatment		
	Day 0	Day 7	Day 28	Day 56
Total sperm number (10^6^ cells)	36.4 ± 4.6 ^a^	15.1 ± 1.1 ^a^	9.5 ± 0.7 ^ab^	1.6 ± 0.2 ^b^
Motile sperm (%)	81.7 ± 1.0 ^a^	23.3 ± 1.9 ^b^	41.7 ± 5.4 ^bc^	8.3 ± 1.9 ^c^
Viable sperm (%)	86.8 ± 0.5 ^a^	64.2 ± 1.5 ^b^	67.1 ± 2.2 ^b^	9.3 ± 1.1 ^c^
Normal head morphology (%)	93.4 ± 0.7 ^a^	87.1 ± 1.4 ^a^	91.0 ± 0.2 ^a^	84.1 ± 0.9 ^a^
Normal tail morphology (%)	88.2 ± 1.9 ^a^	75.4 ± 3.3 ^b^	85.2 ± 0.9 ^ab^	75.8 ± 0.6 ^b^

## Data Availability

The data presented in this study are available on request from the corresponding author.
